# Low-Dose Sulfonylurea Plus DPP4 Inhibitor Lower Blood Glucose and Enhance Beta-Cell Function Without Hypoglycemia

**DOI:** 10.1210/clinem/dgae033

**Published:** 2024-01-24

**Authors:** Ruth L M Cordiner, Khaled Bedair, Andrea Mari, Ewan Pearson

**Affiliations:** Division of Population, Health and Genomics, Ninewells Hospital and Medical School, University of Dundee, Dundee DD1 9SY, UK; Division of Population, Health and Genomics, Ninewells Hospital and Medical School, University of Dundee, Dundee DD1 9SY, UK; National Research Council, Institute of Neuroscience, University of Padua, 35127 Padua, Italy; Division of Population, Health and Genomics, Ninewells Hospital and Medical School, University of Dundee, Dundee DD1 9SY, UK

**Keywords:** sulfonylureas, gliclazide, DPP4 inhibitors, type 2 diabetes, KATP channel, bet-cell function

## Abstract

**Context:**

Low-dose sulfonylureas (SUs) have been found to augment the classical incretin effect, increase glucose sensitivity and late phase incretin potentiation.

**Objective:**

To evaluate potential synergy between low-dose SU plus a dipeptidyl peptidase 4 (DPP4) inhibitor.

**Methods:**

Unblinded randomized crossover study at the Clinical Research Centre, University of Dundee. Thirty participants with T2DM (HbA1c < 64 mmol/mol) were treated with diet or metformin. Participants completed 4, 14-day blocks in a random order: control, gliclazide 20 mg (SU), sitagliptin 100 mg (DPP4 inhibitor [DPP4i]), or combination (SUDPP4i). A mixed meal test was conducted after each intervention. The primary outcome was the effect of treatment on beta-cell glucose sensitivity. Secondary outcomes included frequency of glucose <3 mmol/L on continuous glucose monitoring, subanalyses by genotype (*KNCJ11 E23K*), gender, and body mass index.

**Results:**

SU combination with DPP4i showed additive effect on glucose lowering: mean glucose area under the curve (mean 95% CI) (mmol/L) was control 11.5 (10.7-12.3), DPP4i 10.2 (9.4-11.1), SU 9.7 (8.9-10.5), SUDPP4i 8.7 (7.9-9.5) (*P* < .001). Glucose sensitivity mirrored the additive effect (pmol min^−1^ m^−2^ mM^−1^): control 71.5 (51.1-91.9), DPP4i 75.9 (55.7-96.0), SU 86.3 (66.1-106.4), SUDPP4i 94.1 (73.9-114.3) (*P* = .04). The additive effect was seen in men but not women. Glucose time in range <3 mmol/L on continuous glucose monitoring (%) was unaffected: control 1 (2-4), DPP4i 2 (3-6), SU 1 (0-4), SUDPP4i 3 (2-7) (*P* = .65).

**Conclusion:**

Low-dose sulfonylurea plus DPP4i has a potent glucose-lowering effect through augmentation of beta-cell function. A double-blind randomized controlled trial would formalize efficacy and safety of this combination, which may avoid negative aspects of SU.

Sulfonylureas (SUs) have been utilized in the treatment of type 2 diabetes mellitus (T2DM) for over 70 years (1). However, their use has declined due to their associations with hypoglycemia, weight gain, limited durability, and their lack of positive cardiovascular outcome data compared with newer agents. Currently, international guidelines recommend the use of SUs “if cost is an issue” (2). However, the cost of diabetes care is escalating; the global prevalence of T2DM is predicted to increase from 382 million people to 592 million by 2035, which includes 69% increase in prevalence in developing countries and a 20% increase in developed countries (3, 4). The predicted absolute global economic burden of diabetes care will increase from US$1.3 trillion (95% CI 1.3-1.3) in 2015 to $2.2 trillion (2.2-2.3) in 2030, which translates to an increase in costs as a share of the global GDP from 1.8% (1.7-1.9) in 2015 to a maximum of 2.2% (2.1-2.2) (5). This increase in per capita cost therefore poses a global emergency to control cost. A systematic review indicated that the economic burden of diabetes most directly affects patients in low- and middle-income countries, with the magnitude of cost differing considerably between countries (6). Therefore, there is pharmacoeconomic need to provide cost-effective diabetes care, including how we modernize our use of our cheaper generic therapies such as SUs and dipeptidyl peptidase 4 inhibitors (DPP4is).

Studies in neonatal diabetes mellitus (NDM) have provided insight into the beta-cell response of SUs. Studies in patients with NDM due to activating mutations in *KCNJ11* found that these patients were able to effectively switch from insulin to high-dose SU, with resulting tight glycemic control while avoiding hypoglycemia. Patients with NDM have no insulin response to intravenous glucose but have a significantly increased insulin response to oral glucose or mixed-meal stimulus following SU initiation, suggesting augmentation of the incretin effect (7, 8). In addition, a supra-additive effect of SU in combination with high concentrations of intravenous gastric inhibitory polypeptide (GIP) has also been shown in HNF1A maturity-onset diabetes of the young (9) and in T2DM treated with standard dose glipizide (10). Our previous work has shown that low-dose SUs augment the incretin effect (11). Using isoglycemic clamps in patients with T2DM treated with diet or metformin monotherapy, we demonstrated that a 20-mg dose of gliclazide reduced the mean glucose area under the curve (AUC) during oral glucose tolerance test from 12.0 to 10.8 mmol/L (*P* = .0006), augmented the incretin effect from 35.5% to 55% (*P* = .04), and increased glucose sensitivity by 50% (*P* = .01) and enhanced late phase incretin potentiation (*P* = .04).

Given that we have uncovered a novel mechanism of SUs at low dose that results in glucose-regulated insulin secretion, in part mediated by the incretin effect, we hypothesized that DPP4is, which increase endogenous incretins, would be a potent drug to combine with low-dose SUs. We aimed to explore the efficacy of low-dose SUs and endogenous incretins, as monotherapy, and in combination with a DPP4is on parameters of beta-cell function utilizing multiple mixed-meal tolerance tests (MMTs), beta-cell modeling and continuous glucose monitoring (CGM).

## Materials and Methods

### Recruitment

As this study was a follow-on from our previous work exploring low-dose SUs and the incretin effect, the same recruitment criteria were applied (11). Thirty participants were recruited with physician-diagnosed T2DM treated with diet or metformin monotherapy, HbA1c < 64 mmol/mol, aged ≥ 40 and ≤ 80 years and with renal and hepatic function in the biochemical reference range from local laboratories. To avoid heterogeneity within the cohort, only White British participants were recruited. All participants had the capacity to express informed, written consent. Participants not meeting inclusion criteria were excluded. Patients who were pregnant, lactating, or planning to conceive within the study period were ineligible. Patients participating or who were recruited in a clinical study within the preceding 30 days were also ineligible.

### Study Design

**Figure 1.**
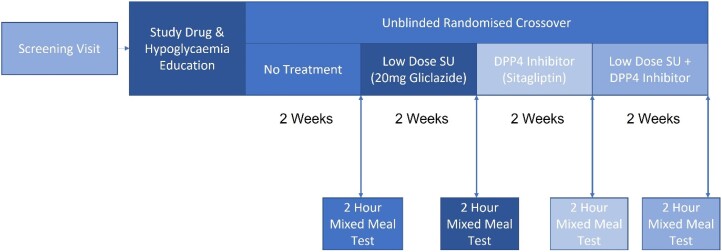
Unblinded, randomized, crossover study design involving 4 different study intervention periods, each 14 days’ duration. Participants completed a 2-hour mixed-meal tolerance test at the end of each study period.

We undertook a single-site, open label, randomized crossover study involving MMT (Fig. 1). The study was approved by the East of Scotland Research Ethics Committee (REC 18/ES/0092) and registered with ClinicalTrials.gov (NCT04192292). All research was conducted in accordance with the Declaration of Helsinki, and informed written consent was obtained for all participants prior to study inclusion.

Study visits took place at the Clinical Research Centre, Ninewells Hospital and Medical School. The study involved 4 intervention blocks, each of 14 days’ duration, to assess response following different combination of low-dose SUs or DPP4is: no intervention (no change to standard care), low-dose SU (20 mg of gliclazide once daily), DPP4i (100 mg of sitagliptin once daily), low-dose SU + DPP4i (20 mg of gliclazide + 100 mg of sitagliptin once daily).

Participants attended the research center on 6 separate visits. A screening visits confirmed eligibility and obtained written informed consent. The second visit provided education regarding study drugs, self-monitoring of blood glucose, and hypoglycemia. The other 4 visits were performed at the end of each block.

Participants underwent a 2-hour MMT at the end of each block. Participants were fasted for 8 hours prior to intervention and all regular medications, including metformin, were withheld until the end of the test. On arrival to the center, a single intravenous cannula was inserted into the participant's arm for blood sampling. For MMT involving study drug, participants took the study drug on arrival at the research center. After 60 minutes, a standard liquid meal (Fortisip Compact, Nutricia, NL) was given. Blood samples for insulin, C-peptide, and glucose were taken at 7 defined time points: 0, 15, 30, 35, 60, 90, and 120 minutes. A single sample for total GLP-1, GIP, and glucagon concentrations was taken at time 0. Plasma concentrations of gliclazide were sampled at time 0, 60, and 120 minutes in all participants (n = 30); 9 participants were consented to complete a prolonged MMT for further sampling at 4, 8, and 24 hours for gliclazide pharmacokinetic profiling. The end of study was determined as last patient last visit.

### Study Drugs

Gliclazide 40-mg tablets were sourced from Alliance Pharmaceuticals (UK) and halved by Tayside Clinical Trials Pharmacy. The DPP4i was sitagliptin in the form of Januvia 100-mg tablets (Merck Sharp and Dohme Ltd, UK). A 100-mg once daily dosing schedule was chosen, as pharmacokinetic studies in healthy individuals showed a more consistent DPP4 inhibition profile over a 24-hour period. In comparison, a 50-mg dose only provided 12 hours of consistent DPP4 inhibition (12).

### Liquid Meal

The liquid meal comprised 160 mL of Fortisip Compact, the nutritional content for the given volume was 184 kcal, protein 15.36 g, carbohydrate 47.52 g, fat 14.88 g, and nil fiber.

### Continuous Glucose Monitors

The Freestyle Libre Pro Flash Continuous Glucose Monitoring System was used throughout study (Abbott).

### Blood Collection

All blood collection was performed utilizing BD Vacutainer systems (Becton, Dickinson and Company, NJ, USA). Samples were iced following collection and centrifuged immediately in accordance with recommended guidance from receiving laboratories.

### Laboratory Analyses

#### **Insulin and** C-**peptide**

Analysis of insulin and C-peptide was performed by Clinical Chemistry, Royal Devon, and Exeter Hospital—602 modules Cobas 8000 automated platform using sandwich chemiluminescence immunoassay (Elecsys insulin, Belgium)

#### Glucose

Glucose analysis was performed by NHS Tayside Blood Sciences at Ninewells Hospital utilizing Siemens ADVIA Chemistry, Glucose Hexokinase_3 Concentrated Reagents (UK).

#### Glucagon

Glucagon analysis was performed by the Immunoassay Core Biomarker Laboratory, University of Dundee, utilizing EMD Millipore glucagon radioimmunoassay kit (Merck, Billerica, MA, USA).

#### Incretins

Total GLP-1 and GIP analyses were performed by the Immunoassay Core Biomarker Laboratory, University of Dundee, utilizing MSD metabolic assay Total GLP-1 and GIP assay (MSD, MD, USA).

#### Gliclazide

Gliclazide analysis was performed by the Biomarker and Drug Analysis Core Facility, University of Dundee utilizing a uniquely developed gliclazide quantification method in human plasma by liquid chromatography separation, and tandem mass spectrometry analysis.

### Data and Statistical Analyses

#### Study outcomes

The statistical analysis is included elsewhere (Supplementary Information (13)). The primary outcome was the change in glucose sensitivity at MMT. Secondary outcomes included the effect of treatment on parameters of beta-cell function, biochemical parameters (glucose, insulin, C-peptide, and incretin hormones), and the pharmacokinetic profile of low-dose gliclazide. The frequency of blood sugar levels <3 mmol/L on CGM, and the effect of *KCNJ11* (E23K) genotype and gender on change in glucose sensitivity with drug treatment were also evaluated.

#### Randomization

Participants were randomized to intervention order using an unblinded web-based randomization software (www.randomisation.com). A copy of the randomization plan was stored in the research center, Tayside Clinical Trials Pharmacy, and the site file.

#### Power

Based on previous data in T2DM, the standard deviation of the difference in AUC glucose between placebo and vildagliptin treatment was 125 mmol/L over 240 minutes (14). With 30 patients, the study would have 80% power (*P* = .05) to detect a difference of one-third of that seen with vildagliptin alone compared with placebo. Similarly, the power would be enough to detect approximately 50% of the difference in AUC_INSULIN_:AUC_GLUCOSE_ ratio seen comparing vildagliptin and placebo.

### Modeling

Two models were developed, a linear mixed-effects model and a generalized additive model, both of which considered the hierarchical nature of the study design of 3 levels: treatment (n = 4), participant (n = 30), and time within the MMT (n = 7 time points per MMT). Participants were randomized to block order and the MMT took place on either day 14, 28, 42, or 56 from the start of study.

#### Linear mixed model with random effect

For the primary outcome a linear mixed-effects model was applied (15) with glucose sensitivity as the dependent variable. Synergy was evaluated via post hoc pairwise comparisons between treatments. In this model, treatment intervention was considered a fixed effect, as was time within the MMT. Intersubject variability, block randomization, and day of MMT were considered as random effects. Finally, Ɛ accounted for all other random effects. All assumptions of linear mixed-effect model residuals were checked for deviation from homoscedasticity or normality.

#### Generalized additive model

As the time course of the insulin, C-peptide, incretin, and glucagon response across the MMT were considered as nonlinear parameters, a generalized additive model was developed (16). In this analysis, “*Treatment*” was considered as a fixed effect whereas “*Time*” was considered as a fixed, nonlinear effect. The model applied smoothing parameters to random effects of intersubject variation, block randomization and day. The model fit was checked using the “*mgcv*” package in R (16).

### Parameters of Beta-cell Modeling

Beta-cell function was assessed using a previously described model (17-19), designed to analyze the MMT tests. The model describes the relationship between insulin secretion and glucose concentration by means of a dose–response function relating the 2 variables and an early secretion component. The dose–response is characterized by its average slope, termed glucose sensitivity, and early secretion by a parameter denoted as rate sensitivity, a marker of early-phase insulin release. The dose–response function is modulated by a time-varying potentiation factor, which accounts for effects of sustained hyperglycemia and incretins. The potentiation factor excursion was calculated as the ration between the values at the end of the 2-hour oral glucose tolerance test and at baseline.

### Data Presentation

Model results are presented in tables of estimates (mean, 95% CI), standard error, test statistic and *P* value. A significant *P* value result provided in the “*Control*” arm represents that the intercept is significantly different to 0. *P* values for treatment interventions demonstrate statistical significance vs control.

### Statistical Software

Data were managed utilizing Microsoft Excel as part of Microsoft Office 365 Pro Plus Version 1908 (Build 11 929.20708). Statistical analysis and graphical presentation were performed in R (20).

## Results

### Baseline Characteristics

Study recruitment ran from September 2019 to September 2020. All study activity was paused due to COVID-19 pandemic between March and August 2020. Two participants withdrew consent for study immediately after screening in March 2020 due to the COVID-19 pandemic; these participants were replaced. One participant withdrew prior to last MMT due to circumstances unrelated to study; data until point of withdrawal were retained as per study consent.

Baseline characteristics of the study cohort (n = 30) were representative of SU users in the Tayside region (Table 1). In a subanalysis by gender, male participants had lower body mass index (BMI) (median; lower quartile, upper quartile) (male 30.5; 25, 33; vs female 39; 31, 41 kg/m^2^, *P* < .001) and were older (male 67.5; 64, 71 vs female 59; 54, 66 years, *P* = .02). The most common concomitant medications were metformin (n = 27), statins (n = 26), proton pump inhibitors (n = 13) and selective serotonin reuptake inhibitors (11).

**Table 1.** Baseline characteristics (median [lower quartile, upper quartile])

Phase of study	Number of participants	Gender (M/F)	Pre-existing treatment (diet/metformin)	Age (years)	Body mass index (kg/m^2^)	Body surface area (m^2^)	HbA1c (mmol/mol)	Duration of diabetes (years)
Full study	30	16/14	3/27	66 (57,70)	32.4 (28.40)	2.1 (1.9, 2.3)	54 (48, 62)	6.5 (4.8, 10.0)
Pharmacokinetic phase	9	3/6	3/6	66 (64, 72)	30.7 (27, 38)	2.1 (1.7, 2.4)	50 (48, 60)	5 (5, 10.5)

Thirty participants were analyzed for the primary outcome of study. Nine participants completed prolonged mixed- meal tolerance tests for 24-hour sampling for low-dose gliclazide pharmacokinetic profiling.

Adverse events included 1 episode of symptomatic hypoglycemia (glucose 3.3 mmol/L), which occurred on DPP4i monotherapy. There were 14 occurrences of detachment of CGM sensors, which were documented as adverse events. Sensors were replaced on the next working day following report to the study team.

### Primary Outcome: Change in Glucose Sensitivity With Treatment.

**Figure 2.**
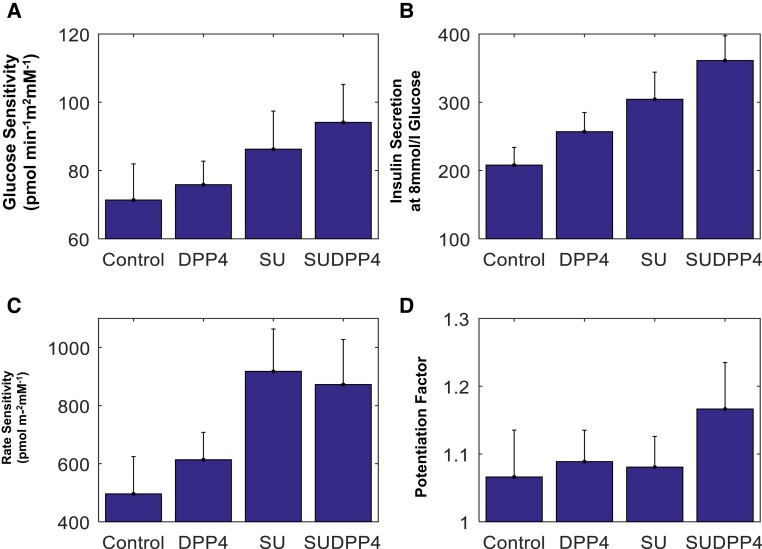
(A) Glucose sensitivity, (B) insulin secretion at 8 mmol/L glucose, (C) rate sensitivity, and (D) potentiation factor by treatment (mean [SEM]).

Linear mixed model parameters of beta-cell function are summarized in Table 2. The plot of glucose sensitivity suggests additive effect of treatment (Fig. 2A). However, the linear mixed-model estimates only show difference in glucose sensitivity with SUDPP4i compared with baseline (*P* = .04) (Table 2).

**Table 2.** Linear mixed effects modeling results of parameters of beta-cell function

Coefficient	Glucose sensitivity (pmol min^−1^ m^2^ mM^−1^)		
	estimates	Standard error	*P* value
Control	71.5 (51.6-91.4)	10.2	
DPP4i	75.9 (34.3-117)	11.0	.70
SU	86.3 (44.7-128)	11.0	.18
SUDPP4i	94.1 (52.6-136)	11.0	.04
**Rate sensitivity (pmol m^2^/mM)**			
Control	494 (220-769)	140	
DPP4i	611 (−32-1260)	188	.53
SU	914 (270-1556)	188	.03
SUDPP4i	879 (234-1522)	188	.04
**Potentiation factor**			
Control	1.07 (0.95-1.19)	0.06	
DPP4i	1.09 (0.81-1.37)	0.08	.79
SU	1.08 (0.80-1.36)	0.08	.86
SUDPP4i	1.17 (0.89-1.45)	0.08	.23

Estimates are shown as mean (95% CI). All values are rounded to 3 significant figures. *P* values for treatment interventions demonstrate statistical significance vs control.

Abbreviations: SU, sulfonylurea; DPP4i, dipeptidyl peptidase 4 inhibitor.

**Figure 3.**
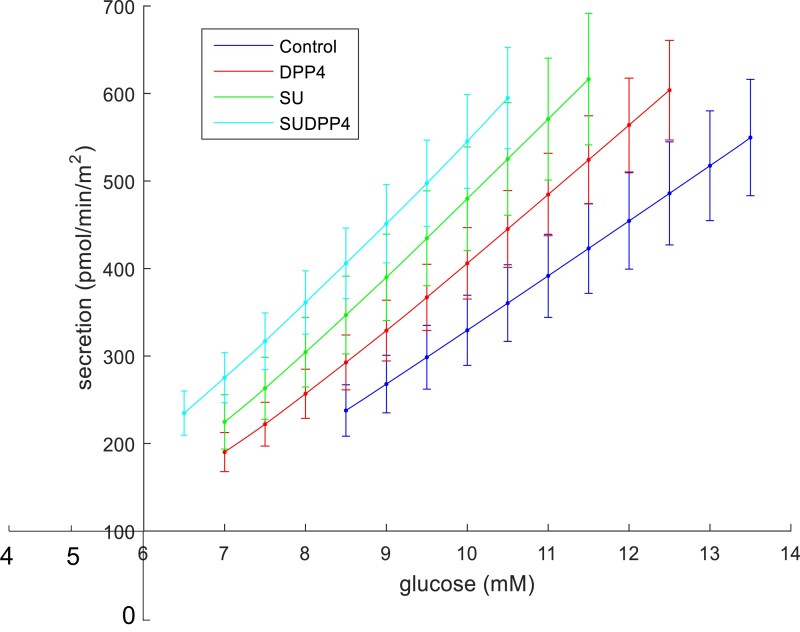
Dose–response by treatment (mean [SEM]).

Figure 3 shows the model-determined relationship between insulin secretion and glucose concentration in each of the 4 intervention groups. A progressive increase in slope is observed across the treatments, representative of the corresponding increase in glucose sensitivity. A left shift is noted in favor of combination treatment in both glucose sensitivity and insulin secretion at 8 mmol/L (Fig. 2B), demonstrating augmentation of beta-cell function at lower glucose concentrations.

### Secondary Outcomes

Rate sensitivity, which is a marker of early insulin release, was augmented by both gliclazide interventions (Fig. 2C and Table 2). This is expected, as gliclazide is a secretagogue influencing early insulin secretion. Predictably, this effect was not further augmented by the combination with DPP4i.

There was no difference in potentiation factor ratio (Table 2). The trend toward an increase in SUDDP4i suggests that had the MMT been prolonged (Fig. 2D), difference may have been observed in late phase potentiation as in our previous study on the incretin effect, which lasted 4 hours (11).

### Glucose

**Figure 4.**
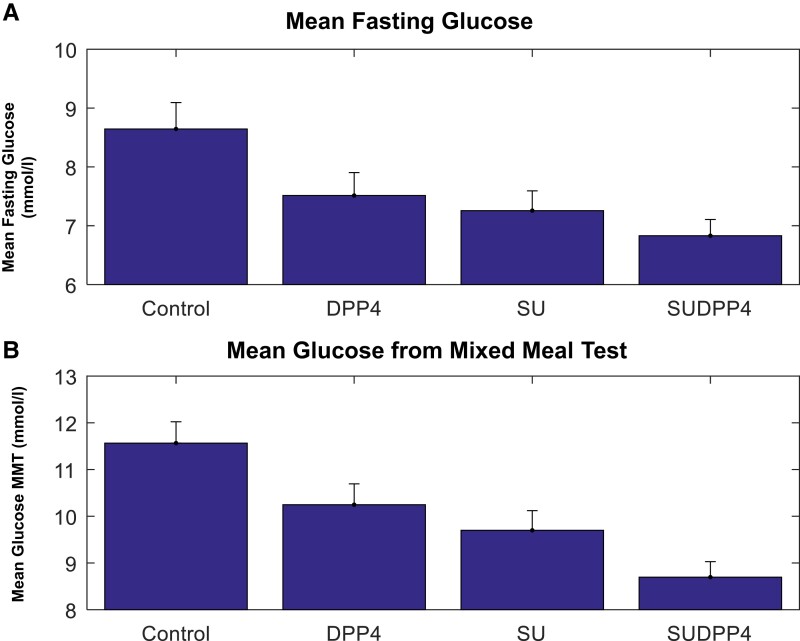
Mean (SEM) (A) fasting glucose and (B) glucose from mixed-meal test.

The mean fasting and mean glucose AUC were reduced in all treatment groups compared with control (Fig. 4). Linear mixed modeling outcomes showed additive effect in terms of difference between estimates of both DPP4i and SU groups vs SUDPP4i (Table 3). SUDPP4i reduced mean glucose AUC compared with both treatments as monotherapy.

**Table 3.** Summary of linear mixed model outcomes for glucose and generalized additive model outcomes for insulin, and C-peptide from mixed meal tolerance test

Coefficient	Mean fasting glucose (mmol/L)			Mean glucose from AUC (mmol/L)		
	Estimates (95% CI)	SE	*P* value	Estimates (95% CI)	SE	*P* value
Control	8.59 (7.88-9.31)	0.37		11.5 (10.7-12.3)	0.42	
DPP4i	7.51 (7.06-8.68)	0.23	<.001	10.25 (8.84-11.7)	0.30	<.001
SU	7.25 (6.09-8.42)	0.23	<.001	9.7 (8.29-11.1)	0.30	<.001
SUDPP4i	6.83 (5.6-8)	0.23	<.001	8.7 (7.29-10.1)	0.30	<.001
	Fasting insulin (pmol/L)			Fasting C-peptide (pmol/L)		
Control	170 (117-224)	27.1		1503 (1271-1735)	118	
DPP4i	137.17 (55.2-219)	14.8	.03	1370 (1016-1725)	62.7	.04
SU	165.46 (83.5-199)	14.8	.76	1495 (1141-1620)	62.7	.9
SUDPP4i	182.24 (99.6-265)	15.1	.42	1615 (1261-1969)	62.7	.07
	Incremental AUC insulin (nmol/L/min^−1^)			Incremental AUC C-peptide (nmol/L/min^−1^)		
Control	41.1 (30.4-51.7)	5.39		144 (111-177)	16.8	
DPP4i	43.0 (18.2-34)	7.56	.71	159 (120-131)	19.8	.45
SU	42.0 (16.4-67.6)	7.55	.90	151 (112-224)	19.8	.72
SUDPP4i	44.3 (18.7-70.1)	7.58	.66	154 (81.2-226)	19.8	.65

Estimates are mean (95% CI). All values are rounded to 3 significant figures. *P* values for treatment interventions demonstrate statistical significance vs control.

Abbreviations: AUC, area under the curve; SU, sulfonylurea; DPP4i, dipeptidyl peptidase 4 inhibitor.

### Insulin and C-peptide

The generalized additive model estimates showed no effect of treatment on either incremental AUC (iAUC) insulin or C-peptide (Table 2). However, when interpreted in context of significant glucose reduction, this would suggest an overall improvement in beta-cell function (Table 3).

### Incretins

As our previous work found that low-dose gliclazide had no impact on dynamic endogenous incretin or glucagon secretion (11), only fasting measurements were performed. Generalized additive model estimates showed that SU reduced fasting GLP-1, GIP, and glucagon concentrations (Table S1 (13)).

### Gliclazide Pharmacokinetics

Study drugs were administered 60 minutes prior to the start of the MMT. Mean gliclazide concentrations (mean [SD]) (ng/mL) were SU 662 (408), SUDPP4i 603 (355) respectively (*P* = .31) and maximum concentrations were 749 (433) and 645 (397) ng/mL (*P* = .01) in the SU and SUDPP4i meal tests, respectively. Combination treatment did not affect the 24-hour profile of gliclazide: mean gliclazide AUC 660 (328) and 535 (223) ng/mL (*P* = .1), maximum concentration 770 (328) and 793 (413) (*P* = .8). Trough concentrations of gliclazide were 370 (183) and 343 (183) ng/mL in the SU and combination groups, respectively.

### Continuous Glucose Monitoring

Blood glucose <3 mmol/L was considered as biochemically significant hypoglycemia in line with the European and American joint position statement (21). Linear mixed-effects modeling of time in range <3 mmol/L on CGM (%) was unaffected: control 1 (2-4), DPP4i 2 (3-6), SU 1 (0-4) (*P* = .64). Only treatments involving SU increased time in range between 3 and 10 mmol/L (%) vs control: control 67.4 (56.6-78.2), DPP4i 64.5 (45.6-83.74), SU 71.83 (52.59-71.25), SUDPP4i 68.4 (66.16-85.83) (*P* < .001 SU and SUDPP4i vs control).

### Effect of *KCNJ11* (E23K) Genotype

**Figure 5.**
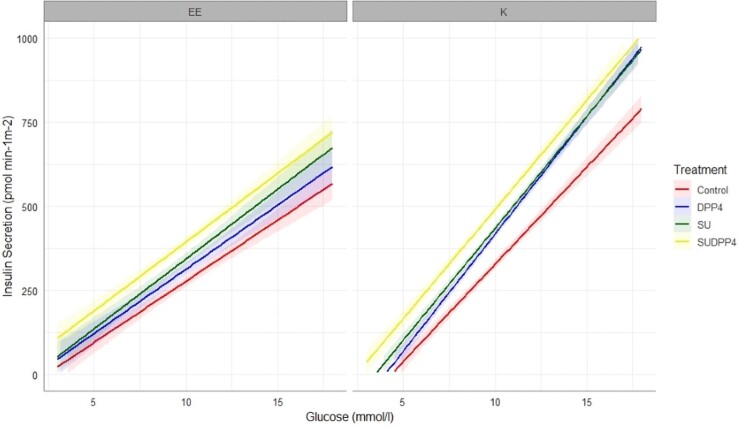
Dose–response to treatment by *KCNJ11 E23K* genotype (mean [SEM]).

The study cohort included 12 EE, 5 KK, and 12 EK heterozygotes, which is slightly higher than the 34% to 48% minor allele frequency reported for Caucasian populations (22). Linear mixed-model estimates showed additive effect of treatment on glucose parameters in EE homozygotes only (Table S2 (13)) and fasting insulin and C-peptide in those carrying the K-allele. Plots of the glucose sensitivity suggest a steeper slope in K-allele carriers (Fig. 5); however, the linear mixed-model estimates were not statistically significant.

### Effect of Gender

**Figure 6.**
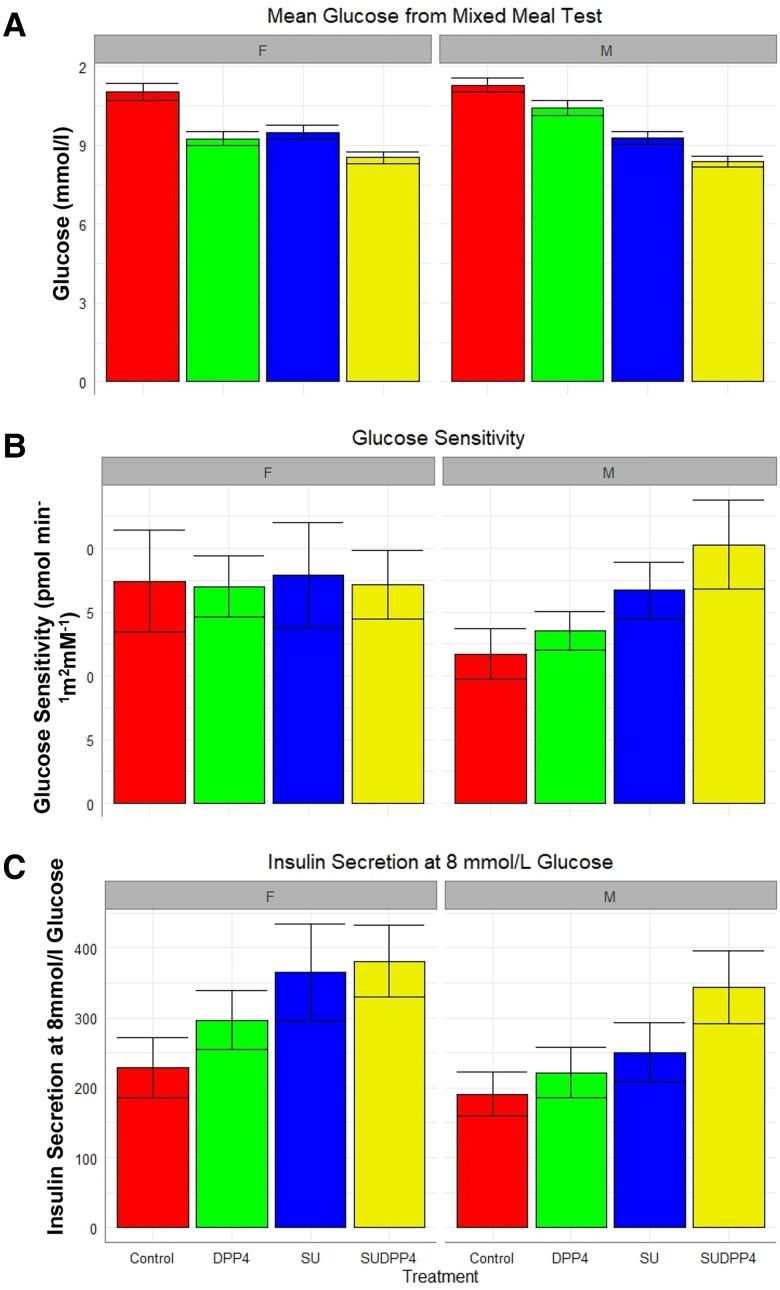
Gender differences in treatment response (mean [SEM]) (A) glucose from mixed-meal test, (B) glucose sensitivity, and (C) insulin secretion at 8 mmol/L glucose.

As previous literature had suggested differing response to SU by sex and BMI (23), the model was adjusted for this interaction. Subanalysis of glucose sensitivity and the dose–response revealed a potent additive effect in male participants, which was not observed in female participants (Fig. 6). Linear mixed modeling estimates suggest that women respond better to DPP4is, which has not been previously documented, with no difference in glucose-lowering effect between DPP4i and SU. In contrast, men showed a greater response to interventions involving SU, including between SU and combination treatment (Table S3 (13)).

## Discussion

The study describes the effect of low-dose SU as monotherapy, or in combination with a DPP4i on parameters of beta-cell function in response to a standardized meal with 4 different interventions: control, 100 mg of sitagliptin, 20 mg of gliclazide, or both. Our results show that combination treatment with SU and DPP4i enhanced glucose control and beta-cell function, without hypoglycemia on CGM, which supports that this combination could have potential use as an effective, low-cost treatment. However, the observed increase in glucose sensitivity with combination treatment was not greater than the sum of the monotherapy responses.

### Low-Dose Sulfonylureas are Potent Glucose-Lowering Agents While Avoiding Hypoglycemia

In this study, we show that 20 mg of gliclazide has potent glucose-lowering potential, which is further enhanced by the addition of a DPP4i while avoiding hypoglycemia. A 2.8 (0.305) mmol/L (mean [SEM]) reduction in mean glucose from AUC was observed with combination treatment vs control. Strikingly, 20 mg of gliclazide as monotherapy was as potent as 100 mg of sitagliptin (mean glucose reduction 1.8 mmol/L (0.3), 1.3 (0.3) mmol/L *P* = .27 gliclazide vs sitagliptin, respectively). A meta-analysis of studies combining standard dose SU with DPP4i has shown a 50% increased risk in hypoglycemia in the first 6 months of treatment (24). We used 20 mg of gliclazide in this study, which showed no difference in the frequency of hypoglycemic events between treatments.

We propose mechanistically that low-dose SUs do not cause hypoglycemia due to their effect on the K_ATP_ channel open state, similar to their mechanism observed in *KCNJ11* neonatal diabetes mellitus (NDM) (25). In *KCNJ11* NDM, high-dose glibenclamide is required to promote insulin secretion and successful transition off insulin (7). Even at these high doses the mutant K channels do not shut completely, resulting in a beta-cell resting membrane potential that is subthreshold for insulin release but primed for other stimuli such as incretins. In T2DM, where there are only minor defects in K_ATP_ channel function, normal doses of SUs fully close the K_ATP_ channels, resulting in insulin secretion despite normal or low glucose. Our findings suggest that a very low dose of SU in T2DM achieves a similar partial closure of the K_ATP_ channels as seen for high dose SU in NDM, working primarily to prime the beta-cell to other secretagogues such as the incretins or amino acids, resulting in glucose regulated insulin secretion and no insulin secretion in the presence of normal or low blood glucose. Therefore, it could be possible to achieve the glycemic benefits of SU, while minimizing negative attributes.

### Combination Low-Dose Sulfonylurea and DPP4i Heightens Parameters of Beta-Cell Function With Additional Effect on Glucose Lowering

Modeling of beta-cell function showed progressive augmentation of the slope of the dose–response (glucose sensitivity) in favor of SUDPP4i; however, although there was a clear additive effect, there was no evident synergy as hypothesized. The relationship of the dose–response is presented in Fig. 3; there are 2 parameters characterizing this relationship. The first is glucose sensitivity, and the second is insulin secretion at fixed glucose concentration, which is equivalent to an intercept. It may be that the left shift in the dose–response may at least in part be independent of glucose sensitivity; however, in this study significant additive effect is also seen in this parameter. It can be postulated that SU in this instance are enhancing insulin section, but maintaining glucose dependence, thus avoiding hypoglycemia, as supported by our CGM findings. The impact of gliclazide on glucose sensitivity has been previously suggested in rat models (26, 27) and in healthy human participants (28), albeit at high dose.

### Gliclazide Pharmacokinetics

This study explored the 24-hour plasma concentration profile of 20 mg of standard release gliclazide, observing trough concentrations of ∼370 ng/mL, mean plasma concentrations of 500-600 ng/mL, and peak of 908 ng/mL. For comparison, an 80-mg dose of gliclazide generates peak plasma concentrations of between 3000 and 5000 ng/mL (29). Interestingly the plasma concentrations observed in this study are only a little lower than those documented for 30 mg of gliclazide MR: trough 472, mean 800 and maximum concentrations of 1100 ng/mL, respectively. A multicenter double-blind randomized controlled trial (RCT) compared the efficacy and safety of gliclazide MR vs glimepiride, in a cohort which included those at higher risk of severe hypoglycaemia (>65 years and renal impairment). Both groups achieved HbA1c reduction of 1.0% with fewer hypoglycemic events with gliclazide MR than glimepiride (3.7 vs 8.9%) (30), which suggests that low-dose modified-release preparations may be preferential in terms safety with similar cost and efficacy to standard-release SU.

### Response by Genotype

In this study, K allele (diabetes risk allele) carriers showed higher fasting glucose, reduced insulin secretion and beta-cell function. Differences were observed in terms of plasma glucose and beta-cell function in carriers of the K allele, but there was no difference in the response to treatment. Plots of the dose–response suggest that K-allele carriers have lower fasting insulin secretion, but in the control group there was a greater slope of glucose sensitivity than EE homozygotes, similar to previous literature (31). However, the difference in slopes with gliclazide treatment was more pronounced in EE homozygotes, suggesting that the 20-mg dose may not be high enough to sufficiently close the K_ATP_ channels in K allele carriers to allow the amplifying pathway to operate. This is supported by a previous study which suggested that K allele carriers require higher doses of SU to achieve glucose reduction, and even higher doses in KK homozygotes (32). A dose–response by genotype study would be required to fully investigate this effect.

### Gender Differences in Response

It is interesting that the additive pattern of glycemic reduction and increase of beta-cell function with SU or in combination with a DPP4i is only seen in men, but not women. However, subanalysis shows the additive effect on insulin secretion at 8 mmol/L glucose is preserved in women, which does suggest effect independent of glucose sensitivity in this instance. These differences in physiological responses by gender mirror findings in large cohort studies (23). In an analysis of subgroups of BMI and gender in patients (n = 22 379) starting SUs or thiazolidinedione in the UK Clinical Practice Data Research Datalink, nonobese males (BMI <30) had a 3.3 mmol/mol better response to SU than thiazolidinedione (*P* < .001); these findings were replicated in the ADOPT study (first-line treatment) (33) and observed in the RECORD study (34). These studies support that there is a sex difference in SU response, and our study provides some physiological insights into these differences. Possible explanations include that the women had higher BMI, although adjusting for BMI does not remove the sex difference (data not shown); or a sex difference in incretin physiology. The ADDITION-PRO study reported that women have greater increased serum GLP-1 concentrations following oral glucose tolerance test than men, even after adjustment for BMI (35). Further studies are warranted to further investigate this.

### Limitations

The main limitation in this study is the wide variability in beta-cell response within a small cohort, which limits power and ability to perform further subanalysis. However, the population would reflect those who would merit second-line intensification in real-world medicine. This open-label physiological study design was adopted as further proof of concept prior to undertaking a formal RCT. The ideal study design would have been a double-blinded RCT, but this was not feasible at this stage.

The advantage of beta-cell modeling is the ability to model static and dynamic parameters of beta-cell function, beyond traditional measures of glucose and insulin secretion. However, as a more complicated procedure, involving multiple parameters it may add some estimation error.

## Conclusion

We have shown that low-dose SUs are potent glucose-lowering agents, which increase the beta-cell dose–response to glucose without increasing hypoglycemia. This response is further augmented in the presence of a DPP4i, although we did not see synergy with this combination. A formal RCT of the efficacy and safety of a low-dose SU in combination with a DPP4i is warranted as a combination treatment may allow modernization of 2 cheap, effective treatments of T2DM with considerable potential for pharmacoeconomic benefit worldwide.

## Data Availability

Some or all datasets generated and/or analyzed during the current study are not publicly available but are available from the corresponding author on reasonable request.

## Clinical Trial Information

NCT04192292 (registered January 31, 2020).

## Abbreviations

AUC: under the curve
BMI: body mass index
CGM: continuous glucose monitoring
DPP4: dipeptidyl peptidase 4
DPP4i: DPP4 inhibitor
GIP: gastric inhibitory polypeptide
MMT: mixed-meal tolerance
NDM: neonatal diabetes mellitus
RCT: randomized controlled trial
SU: sulfonylurea
T2DM: type 2 diabetes mellitus
